# Horizontal Positional Accuracy of Google Earth's High-Resolution Imagery Archive

**DOI:** 10.3390/s8127973

**Published:** 2008-12-08

**Authors:** David Potere

**Affiliations:** Office of Population Research, Princeton University, 207 Wallace Hall, Princeton NJ 08544; E-Mail: dpotere@princeton.edu

**Keywords:** Optical remote sensing, high-resolution imagery, horizontal positional accuracy, land cover validation, Google Earth

## Abstract

Google Earth now hosts high-resolution imagery that spans twenty percent of the Earth's landmass and more than a third of the human population. This contemporary high-resolution archive represents a significant, rapidly expanding, cost-free and largely unexploited resource for scientific inquiry. To increase the scientific utility of this archive, we address horizontal positional accuracy (georegistration) by comparing Google Earth with Landsat GeoCover scenes over a global sample of 436 control points located in 109 cities worldwide. Landsat GeoCover is an orthorectified product with known absolute positional accuracy of less than 50 meters root-mean-squared error (RMSE). Relative to Landsat GeoCover, the 436 Google Earth control points have a positional accuracy of 39.7 meters RMSE (error magnitudes range from 0.4 to 171.6 meters). The control points derived from satellite imagery have an accuracy of 22.8 meters RMSE, which is significantly more accurate than the 48 control-points based on aerial photography (41.3 meters RMSE; t-test p-value < 0.01). The accuracy of control points in more-developed countries is 24.1 meters RMSE, which is significantly more accurate than the control points in developing countries (44.4 meters RMSE; t-test p-value < 0.01). These findings indicate that Google Earth high-resolution imagery has a horizontal positional accuracy that is sufficient for assessing moderate-resolution remote sensing products across most of the world's peri-urban areas.

## 1. Introduction

With more than 200 million users since its release in June 2005 [1], Google Earth (GE) has recently been recognized for its potential to significantly improve the visualization and dissemination of scientific data [2-4]. Yet the imagery which underlies GE has potential applications that extend beyond visualization; the archive could contribute directly to land-cover and land-use change science (LCLUC). GE now hosts high-resolution (< 2.5 meter) imagery from 2000-2008 that spans more than twenty percent of the Earth's land surface, and more than a third of the human population [5]. Imagery at these resolutions allows human observers to readily discriminate between major natural land cover classes and to discern components of the human built environment, including: individual houses, industrial facilities, and roads [6, 7]. Some scientists have recently begun using this rapidly expanding, cost-free imagery source [8-11], but the GE high-resolution imagery archive remains a largely unexploited resource for the scientific analysis and description of the Earth's land surface.

Although high-resolution imagery has long played a role in scientific inquiry, the 1999 and 2001 launches (respectively) of the commercial imaging satellites IKONOS and QuickBird, have generated increased interest in methods that facilitate the efficient extraction of scientifically relevant information from high-resolution imagery. Improvements in algorithm design and computational power have steadily reduced the analytic obstacles for leveraging this imagery, yet the cost of commercial imagery remains prohibitive for many science applications. This cost landscape changed in 2005, when Google began hosting high-resolution commercial imagery at reduced spectral and spatial resolution on its cost-free Google Earth and Google Maps applications.

GE high-resolution imagery does not contain an infrared band and sometimes has a slightly coarser spatial resolution than the native images provided directly from the sensor operators, yet a user of the GE environment is often able to readily discern land cover type, disturbance events, and other relevant attributes based solely on the imagery. Users also have a number of additional resources to rely upon, including: detailed digital elevation models which allow three-dimensional viewing of the imagery, more than five million geo-referenced photos from services such as Panoramio [12], and a rapidly expanding set of vector and image-based overlays from a wide range of commercial geospatial services companies, scientific and government organizations, and millions of individual members of the GE community.

**Figure 1.**
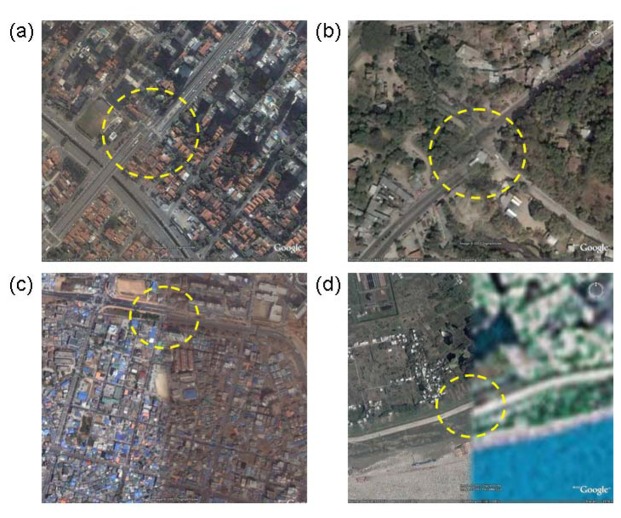
Apparent georegistration problems (highlighted with dashed yellow circles) between adjacent high-resolution images from Google Earth in: (a) Sao Paolo, Brazil, (b) San Salvador, El Salvador, (c) Chonan, South Korea, and (d) Anqing, China. The Anqing example includes both Landsat (right side) and Quickbird imagery (left side).

To make Google's high-resolution imagery as useful as possible, it is necessary to more fully characterize the temporal, spectral, and spatial properties of the archive. Up to this point, Google has been unwilling to release detailed information regarding any of these aspects of their holdings. Of all the desired attributes, georegistration is the most readily tested. Errors in image alignment are apparent in the form of disjoint linear features such as roads and coastlines (Figure 1). In the face of these errors, the question arises: how trustworthy is the horizontal positional information in this archive? Large errors in georegistration would limit the scientific utility of the GE archive. In this analysis, we address this important question of horizontal positional accuracy.

## 2. Data and Methods

The dataset under review in this analysis is the GE high-resolution imagery archive—a global collection of images at roughly 2.5-meter resolution. The bulk of the high-resolution images in GE are from DigitalGlobe's QuickBird satellite, a polar orbiting sensor that produces sub-meter resolution imagery with a horizontal accuracy of 23 meters (90% confidence interval; [13]). Where no high-resolution imagery is available, GE defaults to medium-resolution Landsat imagery. There is no detailed documentation publicly available on the process by which GE ingests high-resolution imagery, however, it is apparent that the overall strategy has been to reproject all images into a geographic (platte carré) projection using the WGS-84 datum. The high-resolution GE imagery is true color, with less dynamic range than the underlying QuickBird, SPOT 5, or aerial photography images. GE's acquisition of high-resolution imagery is a continuous process, with sporadic reports of updates from both Google and the GE user community. There is no meta-data readily available regarding image acquisition dates, spectral transformations, or spatial interpolations. When a GE user zooms into a sufficiently fine level of detail, the name of the imagery provider is sometimes displayed together with a year, but because there is no global vector layer describing scene boundaries, it remains impossible to ascertain acquisition dates with confidence.

To characterize the horizontal positional accuracy of the high-resolution GE archive, we compare the locations of 436 control points in the GE imagery to their equivalent positions in the Landsat GeoCover data set, which has positional accuracy of 50 meters root-mean-squared error (RMSE) [14, 15]. In an ideal assessment of spatial accuracy, we would determine the position of these control points through a global ground-based campaign using global positioning satellites (GPS). However, GeoCover images allow us to more rapidly and inexpensively assess horizontal positional accuracy. In our clustered sampling scheme, we select an average of four points in each of 109 cities. By clustering around cities, we took advantages of GE's city-focused image acquisition strategy. The 109 cities we selected are a sub-set of a pre-existing random 120-city sample that was stratified based on world-region, national income, and city-size [16]. The Landsat imagery for these cities was either provided as part of holdings related to a global study of urban dynamics [16], or was downloaded directly from the Global Land Cover Facility [17]. The GE imagery was downloaded in November 2007.

**Figure 2.**
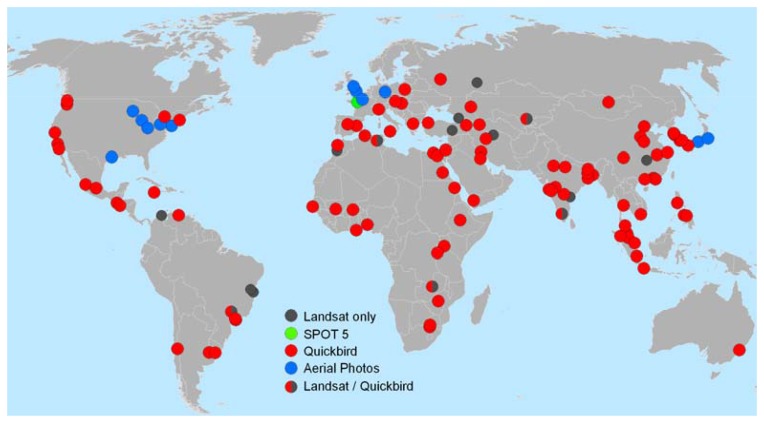
The 120-city global urban sample from Angel *et al.* [16]. Colored circles mark cities where high-resolution Google Earth imagery was available (109 cities) and gray circles mark cities where only Landsat GeoCover base imagery was available (11 cities). Only the 109 cities with high-resolution imagery were included in this study.

The 120 cities that we examined are depicted in Figure 2, where it is clear that 11 cities (9 percent) had only medium-resolution Landsat GeoCover imagery available on GE, and the remaining 109 had high-resolution imagery available for at least half of the city area. Only the 109 cities with high-resolution GE imagery were included in our study. For these cities, we began by identifying control points that could be readily located within the medium-resolution reference images. We generated a GE keyhole markup language (kml) file with the centroids of the 109 cities, and added approximately four control points per city to this kml file using standard GE tools.

The following attributes guided our process for selecting control points: (a) strong vegetative/non-vegetative contrast, (b) large enough to be apparent in 28.5 meter Landsat images, (c) long and linear in form, preferably cross-shaped so that location can be constrained on more than one axis, (d) thin enough to involve only one or two medium-resolution pixels, (e) located within a relatively stable region that would be unlikely to undergo land cover change in the period separating the Landsat and GE image acquisitions. Some examples of useful features include: airport taxiways, road intersections, canal crossings, bridges, and monuments. Features that satisfied most of the criteria, but were avoided because of the potential for land cover change included: agricultural field boundaries, forest logging operations, river features, and new construction events. Once all of the points were added to the kml file, we converted the kml file to an ArcGIS point shapefile.

**Figure 3.**
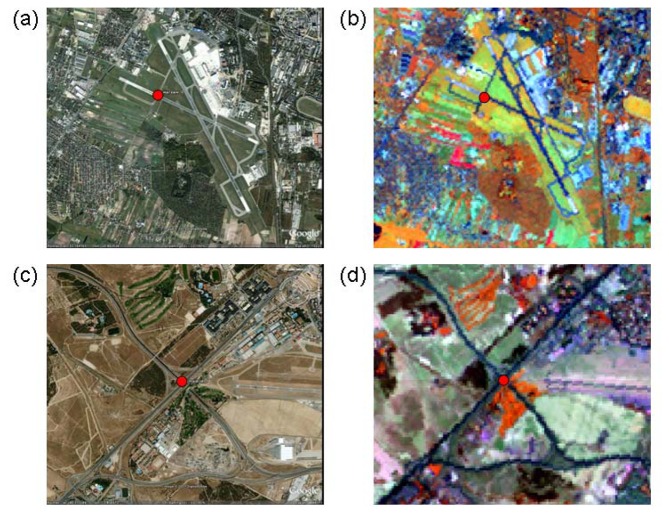
Google Earth (a, c) and Landsat (b, d) control points for an airport in Warsaw, Poland (a, b), and a highway intersection in Madrid, Spain (c, d). Control point pairs appear as red circles; the Warsaw and Madrid windows are 5 km and 3km in width, respectively.

In order to locate these control points within the Landsat GeoCover imagery, we used ArcGIS to view clips of the full Landsat scenes in a 4-5-3 band combination (RGB; Landsat bands 453; near-infrared, short-wave infrared, red) and standard linear stretches (Figure 3). An Arc script linked the view extent of the GE and ArcGIS windows. Control points were identified independently in the Landsat image, added to a shapefile for each city, and re-projected into a geographic projection. These Landsat-derived points were then compared to the equivalent control points from the GE imagery. For each point-pair, an error vector was estimated, with the Landsat control point as the origin and the GE control point as the terminus. For each of these vectors, a direction and magnitude was estimated.

## 3. Results and Discussion

**Figure 4.**
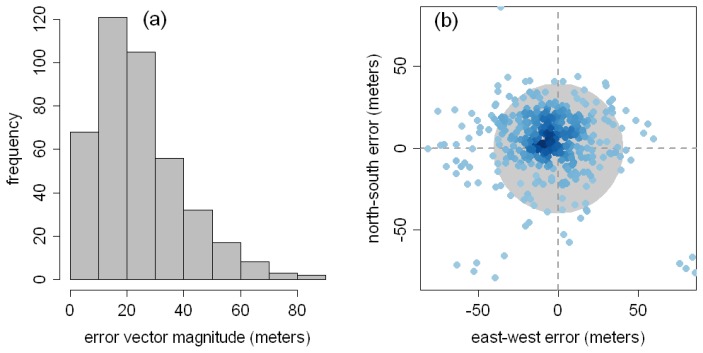
(a) Magnitude of horizontal error vectors for 412 control points (points from six cities with mean errors greater than 1 standard deviation from the global mean are not plotted). (b) Error vectors for 413 control points (23 large-magnitude error vectors are not plotted). The gray crosshair marks the origin of the error vectors (Landsat GeoCover), the blue density-shaded dots are termini (GE), and the gray ring describes the overall accuracy (39.7 meters RMSE).

The overall accuracy of the full sample (436 control points) is 39.7 meters RMSE (Table 1; Figure 4a), with a range of 0.4 to 171.6 meters. The city with the lowest mean offset is Pittsburgh, USA, and the highest is Anqing, China (5.4 and 163.3 meters, respectively). Only five cities contain control points with errors greater than 100 meters. The 48 control points derived from aerial photography have an accuracy of 41.3 meters RMSE, which is significantly poorer than the accuracy of the satellite-derived control points (22.8 meters RMSE; t-test p-value < 0.01). Developed country control points (Europe, United States, Canada, Japan, Australia, and New Zealand) have an accuracy of 24.1 meters RMSE, which is significantly better than the developing country accuracy (44.4 meters RMSE; t-test p-value < 0.01). There are no apparent patterns in the direction of the error vectors (Figure 4b), and no significant correlations between the magnitude or direction of the error vectors and the latitude of the control points. Because details of the GE georegistration process are not publicly available, it is not possible to speculate as to the underlying cause of trends in the magnitude of error vectors. However, these findings indicate that the uncertainty surrounding the position of GE imagery (39.7 meters RMSE) is less than that of the Landsat GeoCover dataset (50 meters RMSE). Conservatively, GE horizontal accuracy is 89.7 meters RMSE (the sum of the Landsat and GE errors), adequate for GE high-resolution imagery to be of use in analyzing a moderate-resolution remote sensing imagery and imagery-derived products.

This geospatial analysis removes one important obstacle to using GE imagery for science applications, yet the spatial resolutions, spectral attributes, and temporal qualities of the GE archive remain uncharacterized. The open nature of the GE archive means that it is possible to build data mining tools that would allow one to map the spatial and spectral properties of the full archive, however there is no similar opportunity for reducing the temporal unknowns. These unknowns in the dates of image acquisitions are of particular concern to the land-cover and land-use change (LCLUC) community. Because the vast majority of Google's high-resolution imagery comes from the QuickBird satellite (launched October 2001), we can place some bounds on image acquisitions, but as time passes this window continues to widen. Unless Google provides vector-based meta-data on their high-resolution GE imagery archive, these unknowns will continue to limit many promising scientific applications.

**Figure 5.**
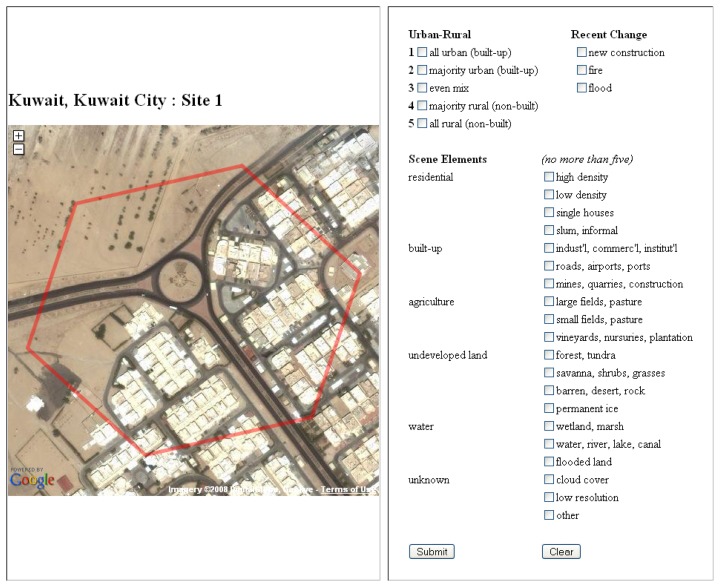
A screen-shot from the Land Cover Validation Tool v 1.0 for a 13-hectare site in Kuwait City, Kuwait. Entries from the right panels are captured in an online database. The imagery is served by the Google Maps application programming interface (API**).**

The Land Cover Validation Tool (LCVT) is one example of the kind of science applications that are made possible with the GE high-resolution archive (Figure 5). LCVT was designed to facilitate the assessment of global land cover and human population maps, the training of supervised land cover classifiers, and the development of land cover change detection algorithms. This web-based application allows users to select a global, random, stratified sample of validation sites. These sites are portrayed with the Google Earth high-resolution imagery archive through the use of the Google Maps Application Programming Interface (API).

In this example, the sites are 13-hectare hexagons (visible in red in Figure 5) that were created using Discrete Global Grid software [18]. There are 100 sites for each of the 120 cities in the Angel *et al.* (2005) global sample, and these sites are stratified based on the fraction of urban area within each hexagon according to a library of Landsat-derived urban maps. Because the tool is web-based, multiple users can simultaneously assess sites using the web-form on the right of Figure 6. User responses are collected in real-time as part of an online database. Although the Google Maps API is an excellent new resource for the global change community, the power of applications such as the LCVT would be greatly enhanced if Google opted to share temporal, spatial, and spectral meta-data on their high-resolution imagery holdings.

## Figures and Tables

**Table 1.** Error vector magnitudes by world region, and globally. ‘Other developed countries’ refers to the United States, Canada, Australia, New Zealand, and Japan.

	**Root Mean Sq. Error (RMSE) (meters)**	**Mean Error (meters)**	**Standard Deviation (σ) (meters)**	**Range (meters)**	**Control Points (N)**	**Sample Cities**
	
**Other Developed Countries**	22.6	19.0	12.3	(0.9 - 53.0)	64	16
**Europe**	25.7	23.1	11.4	(1.6 - 46.0)	60	15
**Latin America and Caribbean**	41.4	30.9	27.9	(0.4 – 111.7)	54	14
**Western and South Central Asia**	42.3	31.5	28.5	(1.4 – 115.0)	78	19
**Southeast and East Asia**	45.9	32.7	32.4	(1.0 – 171.6)	108	27
**Africa**	46.2	35.7	29.6	(2.7 – 160.9)	72	18
	
***total***	***39.7***	***29.4***	***26.6***	***(0.4* – *171.6)***	***436***	***109***
